# Specific microtubule-depolymerizing agents augment efficacy of dendritic cell-based cancer vaccines

**DOI:** 10.1186/1423-0127-18-44

**Published:** 2011-06-20

**Authors:** Chih-Chun Wen, Hui-Ming Chen, Swey-Shen Chen, Li-Ting Huang, Wei-Ting Chang, Wen-Chi Wei, Li-Chen Chou, Palanisamy Arulselvan, Jin-Bin Wu, Sheng-Chu Kuo, Ning-Sun Yang

**Affiliations:** 1 Graduate Institute of Pharmaceutical Chemistry, China Medical University, Taichung, Taiwan; 2 Agricultural Biotechnology Research Center, Academia Sinica, Taipei, Taiwan; 3 Department and Institute of Pharmacology, National Yang-Ming University, Taipei, Taiwan; 4 Department of Allergy and Immunology, IgE Therapeutics, Inc., San Diego, CA, USA; 5 Department of Molecular Biology, The Scripps Research Institute, San Diego, CA, USA; 6 Department of Food and Nutrition, Providence University, Taichung, Taiwan; 7 Department of Marine Biotechnology and Resources, National Sun Yat-Sen University, Kaohsiung, Taiwan

**Keywords:** immunogenic cell death, colchicine, 2-phenyl-4-quinolone, dendritic cells, cancer vaccine

## Abstract

**Background:**

Damage-associated molecular patterns (DAMPs) are associated with immunogenic cell death and have the ability to enhance maturation and antigen presentation of dendritic cells (DCs). Specific microtubule-depolymerizing agents (MDAs) such as colchicine have been shown to confer anti-cancer activity and also trigger activation of DCs.

**Methods:**

In this study, we evaluated the ability of three MDAs (colchicine and two 2-phenyl-4-quinolone analogues) to induce immunogenic cell death in test tumor cells, activate DCs, and augment T-cell proliferation activity. These MDAs were further evaluated for use as an adjuvant in a tumor cell lysate-pulsed DC vaccine.

**Results:**

The three test phytochemicals considerably increased the expression of DAMPs including HSP70, HSP90 and HMGB1, but had no effect on expression of calreticulin (CRT). DC vaccines pulsed with MDA-treated tumor cell lysates had a significant effect on tumor growth, showed cytotoxic T-lymphocyte activity against tumors, and increased the survival rate of test mice. In vivo antibody depletion experiments suggested that CD8^+ ^and NK cells, but not CD4^+ ^cells, were the main effector cells responsible for the observed anti-tumor activity. In addition, culture of DCs with GM-CSF and IL-4 during the pulsing and stimulation period significantly increased the production of IL-12 and decreased production of IL-10. MDAs also induced phenotypic maturation of DCs and augmented CD4^+ ^and CD8^+ ^T-cell proliferation when co-cultured with DCs.

**Conclusions:**

Specific MDAs including the clinical drug, colchicine, can induce immunogenic cell death in tumor cells, and DCs pulsed with MDA-treated tumor cell lysates (TCLs) can generate potent anti-tumor immunity in mice. This approach may warrant future clinical evaluation as a cancer vaccine.

## Background

Cancer vaccines seek to treat malignancies by approaches that induce presentation of tumor-associated antigens (TAAs) in contexts that elicit potent CD4^+ ^and CD8^+ ^T-cell responses and break the tolerance of the host immune system to tumor growth [1,2]. Immunity, including innate immunity and antigen-specific adaptive immunity, and tolerance toward tumors is orchestrated by a network of antigen-presenting cells, the most crucial of which are dendritic cells (DCs) [3,4]. Although clinical trials based on DC-based vaccines have been initiated for certain malignancies [5], unlike in pathogen infection, activation of DCs in tumor microenvironments is weak and ineffective [1,6]; therefore, the development of DC-based vaccines that can not only induce powerful activation of DCs, but also enhance tumor-specific immunity by breaking tolerance is a challenge.

Most anticancer chemotherapeutics influence both tumor cells and the associated immune systems [7]. However, the mechanisms that underlie the various cellular activities that induce immune response, and whether specific necrotic or apoptotic cells are immunogenic or tolerogenic, are still unclear [8,9]. A key-lock paradigm has been proposed to explain the relationship between immunogenic cell death and DCs [9]. Immunogenic cell death is characterized by expression of calreticulin (CRT) and then heat shock proteins (HSPs), HSP70 and HSP90 on the cell surface, and the release of high-mobility group box 1 (HMGB1) proteins. CRT, HSPs and HMGB1 can function as immunological adjuvants for phagocytosis, cross presentation of tumor-derived antigens and antigen processing and presentation by DCs [10]. It is, therefore of interest to investigate the molecular and cellular behaviors of these immunogenic cell death-associated proteins in tumor cell lysates (TCLs) with an eye toward improving the efficacy of TCL-pulsed DC-based vaccines.

A cornucopia of chemotherapeutic drugs including microtubule-targeted agents can induce cell death [11]. These can be classified mainly into two groups [12]: microtubule-depolymerizing agents (MDAs), including vinca alkaloids and colchicine, which inhibit tubulin polymerization; and microtubule-polymerizing agents such as paclitaxel, which stimulate tubulin polymerization. Recent studies indicate that functional microtubules are required for antigen processing by DCs [13] and microtubules mediate NF-κB activation in the TNF-α signaling pathway [14]. Furthermore, in terms of activation of DCs, colchicine was found not only to trigger the maturation of DCs *in vitro *and *in vivo *[15], but also promote antigen-cross presentation by murine DCs [16]. On the other hand, with respect to enhancement of antitumor effect, vincristine increased the antitumor effect of DC-based immunotherapy [17], and was also suggested to enhance the immunogenicity of chronic lymphocytic leukemia cells [18]. Taken together, these findings suggest that MDAs may be able to enhance tumor-specific immune response generated by activation of DCs and induce immunogenic cell death. Both, therefore, could conceivably be used in cancer immunotherapy approaches.

Numerous dendritic cell-based strategies have been employed for developing anti-cancer vaccines, including defined peptide-loaded DCs [19,20], genetically-modified DCs [21,22], DC-derived exosomes [5], apoptotic cell-loaded DCs [23], and tumor cell lysate (TCL)-pulsed DCs [19,24,25]. The possible advantage of the use of whole cell lysates as the source for vaccination is that a full complement of TAAs, including both MHC class I and class II-restricted tumor associated epitopes may be provided, thus reducing the possibility of immune escape by antigen loss variants [26]. A common technique used to generate TCLs is removal of the solid cellular debris by centrifugation after repeated cycles of freezing and thawing of target tumor cells [19,26,27]. The protective effect of DCs pulsed with TCLs has been proven in animal models [24,27], and reported in a range of clinical trials [19,25,28]. Nonetheless, the modest activities reported for TCL-pulsed DC-based vaccines so far need to be further improved and optimized, either in the presence or absence of DC-maturation stimuli [25,28].

In this study, we evaluated the effect of colchicine and two 2-pheny-4-quinolone analogues, which, as MDAs, were shown to confer antitumor effects via inhibition of tubulin polymerization [29-31] on the activation of DCs and subsequent T-cell proliferation. We also investigated the effect of these MDAs on mediating immunogenic cell death in treated B16 melanoma cells via induction of DAMP stress proteins. Possible use of the test MDAs as adjuvants for use in TCL-pulsed DC vaccines was also evaluated. Our findings showed that 2-pheny-4-quinolone analogues could induce phenotypic maturation of DCs. These MDAs were able to augment CD4^+ ^and CD8^+ ^T cell proliferation. TCLs generated from MDA treatment were further able to improve the efficacy of TCL-pulsed DC vaccines. Our findings thus provide useful information for potential clinical application of MDAs in chemotherapy or cancer immunotherapy.

## Methods

### Chemicals and reagents

2-(3-chlorophenyl)-6,7-methylenedioxyquinolin-4-one (CMQ) and 2-(3-fluorophenyl)-6,7-methylenedioxyquinolin-4-one (FMQ) were synthesized as previously described [32]. Each compound was dissolved in dimethyl sulfoxide (DMSO) to obtain a stock solution, and a final concentration of 0.1% DMSO was used in the cell assays. RPMI 1640 medium, DMEM, fetal bovine serum (FBS), penicillin, streptomycin, and all other tissue culture reagents were obtained from GIBCO/BRL Life Technologies (Grand Island, NY) unless otherwise indicated. Colchicine, DMSO, and thiazolyl blue tetrazolium bromide (MTT), and other chemical agents were purchased from Sigma (St Louis, MO). The antibody to β-actin and HRP-labeled anti-mouse and anti-rabbit IgGs were obtained from Santa Cruz Biotechnology Inc. (Santa Cruz, CA). Antibodies to HSP70, calreticulin, HMGB1 and survivin were purchased from Cell Signaling Technologies (Boston, MA). The antibody to glypican-3 was purchased from Abcam (Cambridgeshire, UK), and the antibody to HSP90 was purchased from Millipore (Billerica, MA),

### Mice

Male C57BL/6JNarl mice (6-8-weeks old) were purchased from the National Laboratory Animal Breeding and Research Center, Taipei, Taiwan. All mice were maintained in a laminar airflow cabinet in a room kept at 24 ± 2°C and 40-70% humidity with a 12 h light/dark cycle under specific pathogen-free conditions. All facilities were approved by the Academia Sinica Institutional Animal Care and Utilization Committee, and all animal experiments were conducted according to institutional guidelines.

### Cell lines

The mouse B16F10 (B16) melanoma cells were obtained from American Type Culture Collection (ATCC; Manassas, VA, USA). Cell cultures were maintained in Dulbecco's modified Eagle's medium (DMEM) with 1.5 g/l sodium bicarbonate, 10% fetal bovine serum (FBS), 100 mg/ml streptomycin and penicillin, and 2 mM L-glutamine. Mouse bone marrow cells and bone marrow-derived dendritic cells (BMDCs) were cultured in RPMI 1640 containing 10% FBS, 50 mM 2-mercaptoethanol, 100 mg/ml streptomycin and penicillin, and 2 mM L-glutamine.

### Cell viability determined by MTT assays

B16F10 cells (1 × 10^4^/well) were grown in 96-well plates in DMEM supplemented with 10% FBS in a 5% CO_2 _incubator at 37°C. After incubation for 16 hours, culture media were removed and treated with 9 specified concentrations of CMQ-1 (0.001-10 mM) for 24, 48 and 72 hours. Test culture medium was then replaced with 100 μl culture medium containing 3-(4,5-dimethylthiozole-2-yl)-2,5-biphenyl tetrazolium bromide (MTT) at a concentration of 0.5 mg/ml per well for 4 hours, and then light absorbance was measured with a spectrophotometer at 570 nm. Cell viability was expressed as percentage of vehicle control cells (containing DMSO 0.1% as 100%) cultured in the absence of any test compounds.

### Preparation of tumor cell lysates (TCLs)

B16 cell lysates were prepared as described previously with slight modification [33]. Cells (5 × 10^6^) were seeded onto a 15-cm dish maintained for 16 hours, and then treated with DMSO (0.1%), doxorubicin (2.5 μM), colchicine (2.5 μM), CMQ (2.5 μM) or FMQ (2.5 μM) for 24 hours. After scraping, centrifuging and rinsing twice with PBS, cells were suspended at a concentration of 1 × 10^7 ^cell/ml in PBS, frozen in liquid nitrogen for 2 minutes. Cells were then thawed in a 37°C water bath for 4 minutes and sonicated for 4 minutes to further disrupt the cell suspension. The freeze-thaw-sonicated cycle was repeated for four times in rapid succession. The suspension was then centrifuged at 17,000 × g for 15 minutes and the supernatant (tumor cell lysate) was stored at -80°C.

### Western blot analysis

Tumor cell lysate samples were prepared as previously described. In order to determine the expression levels of DAMPs and tumor-associated antigens, B16 tumor cells were harvested after treatment with indicated test compounds, followed by four freeze-thaw cycles. Samples were subsequently resolved by SDS-PAGE using 8%, 10% or 15% gels. The resolved proteins were transferred to a PVDF Immobilon-P membrane (Millipore, Bedford, CA.), and the membrane was blocked with 5% non-fat dry milk in PBST buffer [phosphate-buffered saline (PBS) containing 0.1% Tween 20] for 60 minutes at room temperature. The membranes were then incubated overnight at 4°C with commercially available antibodies (1:1000 dilutions). Loading of equal amounts of protein was assessed using mouse β-actin. The blots were rinsed three times with PBST buffer for 5 minutes. Washed blots were incubated with HRP-conjugated secondary antibody (1:10,000 dilution) and then washed again three times with PBST buffer. The transferred proteins were visualized with an enhanced chemiluminescence (ECL) detection kit (Amersham Pharmacia Biotech, Buckinghamshire, UK). Quantification of bands was performed using Image J software.

### Generation of mouse bone marrow-derived dendritic cells (BMDCs)

BMDCs were generated as previously described with slight modification [34]. Briefly, on Day 0, the bone marrow was collected from femurs and tibiae after euthanasia and then flushed with RPMI-1640 medium using a syringe with a 0.45-mm needle. Red blood cells in suspension were lysed with ACK lysing buffer (150 mM NH_4_Cl, 1.0 mM KHCO_3_, 0.1 mM EDTA) for 5 min. Bone marrow cells were suspended at a density of 1 × 10^7^cells/30 ml in RPMI-1640 containing 10% FBS, 2 mM L-glutamine, 1% of nonessential amino acids and 100 U/mL penicillin and 100 μg/mL streptomycin) supplemented with 20 ng/mL of GM-CSF (Peprotech, Rocky Hill, NJ) in 15-cm dishes at 37°C with 5% CO_2_. On day 2, two-thirds of the medium was removed and 30 mL fresh medium with GM-CSF was added to the cells. On day 5, culture plates were gently swirled and the floating and loosely adherent cells were discarded. Aliquots of 75% culture media were replenished with 20 ng/mL GM-CSF (PeproTech EC, London, UK) and 20 ng/mL IL-4 (Peprotech, Rocky Hill, NJ). On day 7, non-adherent cells were collected and used as the immature DC population for subsequent tests and analyses. More than 92% cells were CD11c^+ ^as measured by flow cytometry.

### Dendritic cells pulsed with tumor cell lysates

DCs were suspended in RPMI-1640 medium at a concentration of 2 × 10^6 ^cells/3 ml in 6-well plate. Tumor cell lsysates (TCLs, 400 μg) from the various treatments described in "Preparation of tumor cell lysates (TCLs)" above were added to the DC culture for 12 hours and then 1 μg/ml of lipopolysaccharides (LPS) was added as a maturation stimulus. After incubation with LPS for 12 hours, TCLs-pulsed DCs were harvested as DC vaccines for immunization.

### Different culture conditions for tumor cell lysate-pulsed DCs

DCs generated from bone marrow were incubated under culture conditions with a regimen of pulsing and stimulating period: (i) without GM-CSF (20 ng/ml) and IL-4 (20 ng/ml), (ii) GM-CSF (20 ng/ml), (iii) GM-CSF (20 ng/ml) and IL-4 (20 ng/ml). These DCs were then further treated with: vehicle only without any treatment (imDCs), LPS for 24 hours (maDCs), TCL plus LPS for 24 hours (CTCL-CMQ) or TCL for 12 hours and then LPS for 24 hours (TCL-CMQ).

### Measurement of expression of IL-12p70 and IL-10

Expression of IL-12p70 and IL-10 were measured using a commercial ELISA kit (R&D Systems, Minneapolis, MN) according to the manufacturer's instructions. Briefly, flat-bottomed 96-well plates were coated with capture antibodies, incubated with samples for 2 hours, washed four times with PBS, developed with appropriate biotinylated secondary antibodies for 2 hours and washed a further four times with PBS. Then plates were incubated with streptavidin-HRP conjugates for 30 minutes, washed five times, TMB substrate solution was then added and stopped by 0.2 M sulfuric acid. The OD values were measured at 450 nm.

### *In vivo *B16 melanoma tumor model

For the tumor challenge, B16 tumor cells were collected at 80% confluence, washed, resuspended in PBS, and injected subcutaneously (10^5 ^cells/50 μl/mouse) into the right flanks of mice. On day 8 post-tumor cell inoculation (when the tumor volume reached 50-80 mm^3^), test mice were vaccinated with different preparations of TCL-pulsed DCs (5 × 10^5 ^cells/50 μl/mouse) by intratumoral injection. C57BL/6 mice were divided into seven experimental groups (eight mice per group). The seven treatments were: (i) PBS (control, Ctrl), (ii) mature DCs (maDC), (iii) TCL-DMSO, (iv) TCL-DX, (v) TCL-C, (vi) TCL-CMQ, (vii) TCL-FMQ. These vaccination sets were used for priming and booster vaccination of mice. Two boosters were performed, one on day 10 and one on day 13. Ten days after the second booster (on day 23), splenocytes were harvested from immunized mice and assayed for cytotoxic T lymphocyte (CTL) activity. Tumor volumes were determined from the length (L) and width (W) of test tumors, as measured with a caliper in a blinded manner, by the formula: V = L × W^2^/2. Survival of mice was recorded over 40 days following tumor challenge.

### Tumor cell lysis by cytotoxic T lymphocyte (CTL)

Cytotoxicity assays for specific cell lysis were performed using the DELFIA EuTDA cytotoxicity method [35]. B16 cells were trypsinized and suspended at a of density of 1 × 10^6 ^cells/ml, then 5 μl BATDA labeling agent was added to each 4 ml of cells for 15 minutes at 37°C. After labeling, cells were centrifuged, washed in PBS, and resuspended at a density of 5 × 10^4 ^cells/ml in DMEM. Next, 5 × 10^3 ^BATDA-labeled B16 target cells in 100 μl of medium were plated into each well of 96-well V-bottomed plates. Splenocytes (effector cells) from vaccinated animals 7 days after second boosting were added to the target cells with ratios ranging from 1:5 to 1:80 for 3 hours at 37°C. Conditions were also established to measure the background level, spontaneous release and maximal lysis. After incubation and centrifugation, 20 μl of supernatant containing released BATDA was transferred to 200 μl of europium solution in a 96-well flat-bottomed plate which was shaken for 15 minutes at 25°C. Plates were analyzed on a time-resolved DELFIA fluorometer (Wallac Victor^3^, Perkins- Elmer, Shelton, Connecticut, USA). Percentage of specific cell lysis was calculated as follows: (experimental release - spontaneous release)/(maximum release - spontaneous release) × 100. Maximum release was determined from 5 × 10^3 ^labeled target cells lysed with DELFIA lysis buffer (Perkin-Elmer) in triplicate wells. Spontaneous release was measured by incubating 5 × 10^3 ^target cells in the absence of effector cells in triplicate wells. Results are reported as mean values of triplicate wells.

### *In vivo *depletion of immune cell subsets

*In vivo *Ab ablation of rat anti-CD4 (GK1.5), anti-CD8 (53-6.7), and anti- NK1.1 (PK136) monoclonal antibodies (100 μg/injection/mice) (all from BioLegend, San Diego, CA) were performed by intraperitoneal injection to deplete CD4^+ ^T cells, CD8^+ ^T cells, and NK cells, respectively, on day 1 before vaccination and on days 2, 5 and 8 after tumor challenge. Normal rat IgG (Sigma) was used as a negative control. C57BL/6 mice (*n *= 5) were immunized by intratumoral injection with DC-based vaccine pulsed with CMQ-treated TCLs on days 7, 10 and 13 after tumor challenge. Survival of mice was observed up to 40 days after tumor challenge.

### Different delivery systems of DC-based vaccines pulsed with CMQ-treated tumor cell lysates

C57BL/6 mice (*n *= 6) were immunized by intratumoral, intranodal, or subcutaneous injection with DC-based vaccines pulsed with CMQ-treated TCLs on days 7, 10 and 13 after tumor challenge. One week after immunization, right flanks of mice were subcutaneously inoculated with B16 tumor cells (10^5 ^cells/50 μl/mouse). Tumor volumes were determined from the length (L) and width (W) of test tumors, as measured with a caliper in a blinded manner by the formula: V = L × W^2^/2. Survival of mice was recorded over 44 days following tumor challenge.

### Analysis of DC phenotype

DCs were harvested and washed in staining buffer (sterile PBS, 1% FBS) before addition of antibodies. Nonspecific binding was blocked with anti-CD16/CD32 (BD Pharmingen, San Diego, CA) for 15 minutes at 4°C. Cells were then stained with anti-CD11c-phycoerythrin (PE) (BD Pharmingen, San Diego, CA) and related phenotypic maturation markers of DCs [anti-CD40-fluorescein isothiocyanate (FITC), anti-CD80-FITC, anti-CD86-FITC, and anti- I-A/I-E -FITC, all from BD, Pharmingen]. Cells were incubated for 30 minutes at 4°C before washing with staining buffer twice. Cells were then resuspended and fixed in 200 mL 2% formaldehyde solution before analysis with a FACS LSR2 flow cytometer using DIVA software (BD Biosciences, San Diego, CA).

### Mixed lymphocyte reaction induced by DCs

Responder CD4^+ ^T cells and CD8^+ ^T cells, used for the allogeneic T-cell reaction, were isolated by being passed through mononuclear cells in a MACS column (Miltenyi Biotec, Auburn, CA). DCs were treated for 24 hours with CMQ (0.1, 0.5 and 1 μM) or colchince (2.5 μM). After harvesting, DCs (5 × 10^3 ^cells) were added to 1 × 10^5 ^allogeneic T cells in flat-bottomed 96-well microtiter culture plates. During the last 16 of the 72 hours of culturing, proliferation of T cells was determined using a 5-bromo-2-deoxyuridine (BrdU)-based Cell Proliferation ELISA kit (Roche, Heidelberg, Germany) according to the manufacturer's instructions. The T-cell proliferation was expressed as the stimulation index: the OD_450 _value of co-culture of treated-DCs and T cells divided by the value of co-culture of DMSO-treated-DCs co-cultured with T cells. Results are presented as means of the values of triplicate cultures.

### Statistical analysis

Data are presented as mean ± SEM or ± SD. Statistical analyses were carried out with GraphPad Prism 5.0 (San Diego, CA). Statistical difference between groups was compared by Student's *t*-test. Differences in survival time and rate were evaluated by a log-rank (Mantel-Cox) test of the Kaplan-Meier survival curves. All statistical tests were two-sided. P values less than 0.05 were considered statistically significant (*, *P *< 0.05; **, *P *< 0.01; ***, *P *< 0.001).

## Results

### Effect of various microtubule-depolymerizing agents on growth of B16 melanoma cells

**Figure 1:**
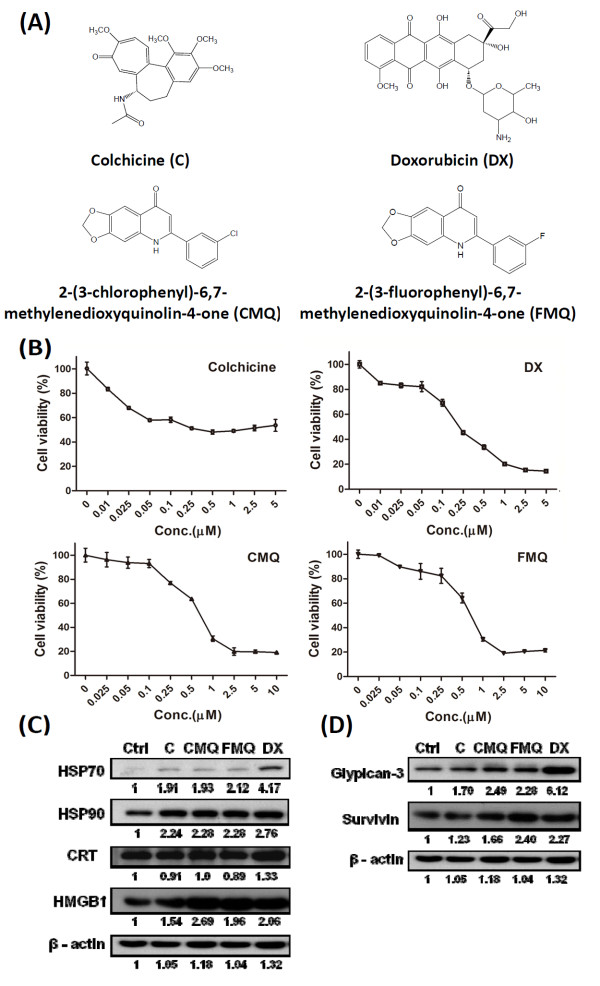
**Expression of damage-associated molecular patterns (DAMPs) and tumor-associated antigens in tumor cell lysates of treated B16 melanoma**. (A) The chemical structures of microtubule-depolymerizing agents and doxorubicin tested in this study are shown in A. (B) Effect of MDAs and doxorubicin on cell viability of B16 melanoma cells. Mouse B16 melanoma cells were treated with the indicated concentrations of colchicine, doxorubicin (DX), 2-(3-chlorophenyl)-6,7-methylenedioxyquinolin-4-one (CMQ) and 2-(3-fluorophenyl)-6,7-methylenedioxyquinolin-4-one (FMQ) for 24 h, as shown in B. The values are represented as the percentage of viable cells; the vehicle control group was regarded as 100% viable. Cell viability was determined by MTT assay and data were expressed as mean ± S.D. for triplicate culture samples. (C&D) Effect of MDAs and doxorubicin on expression of damage-associated molecular patterns (DAMPs) and tumor-associated antigens in treated B16 melanoma cells. B16 tumor cells were treated for 24 h with vehicle control, colchicine (C), CMQ, FMQ or DX, at a concentration of 2.5 μM. After treatment, TCLs were obtained through four freeze-thaw cycles. Western blot analysis for protein expression of damage-associated molecular patterns (DAMPs), heat shock protein 70 (HSP70), heat shock protein 90 (HSP90), calreticulin (CRT) and high-mobility group box-1 (HMGB1), is shown in C, and expression of tumor-associated antigens including glypican-3 and survivin, is shown in D. Expression of β-actin was used as an internal control. The results show one representative experiment of three independently performed experiments.

Several 2-phenyl-4-quinolone derivatives including CMQ and FMQ have been shown to confer anti-tumor activity via the mitochondria (intrinsic pathway), death receptors (extrinsic pathway), or endoplasmic reticulum (ER) stress [31,36,37]. To evaluate whether CMQ and FMQ and colchicine (chemical structures shown in Figure 1A) as microtubule-depolymerizing agents, can cause cell death leading to immunogenic cell death, we examined their effect along with the effect of doxorubicin as positive control on cell proliferation of B16 melanoma cells. Twenty-four hours post treatment, colchicine effectively inhibited approximately 50% of test cell growth at concentrations greater than 0.05 μM. CMQ and FMQ exerted similar inhibitory patterns in a dose-dependent manner at concentrations from 0.1 to 2.5 μM, and inhibition reached a plateau at approximately 2.5 μM (Figure 1B). Doxorubicin inhibited tumor cell growth at all the concentrations tested in this study. Our findings suggest that CMQ and FMQ conferred a similar cytotoxic effect on B16 tumor cells growth to doxorubicin. Colchicine apparently exerts different cytotoxic patterns on growth of B16 melanoma cells.

### Effect of specific microtubule-depolymerizing agents on expression of immunogenic cell death-related proteins and tumor-associated antigens

Recent studies have revealed that increased expression of DAMPs, a characteristic of immunogenic cell death, can increase immunogenicity in cancer vaccines via ER stress or other signaling pathways [38-40]. Whether microtubule-depolymerizing agents can induce DAMPs has not, to our knowledge, been previously reported. Therefore, we measured the expression of several DAMPs (HSP70, HSP90, CRT and HMGB1) in TCLs from B16 cells after treatment with 2.5 μM colchicine, CMQ, FMQ or doxorubicin. The MDAs colchicine, CMQ and FMQ induced the expression of HSP70, HSP90 and HMGB1 in tumor cells as compared with cells treated with vehicle control, but did not affect the expression of CRT (Figure 1C). Doxorubicin induced higher levels of expression of the same DAMPs than the MDAs tested. Specific tumor-associated antigens including glypican-3 and survivin are markers for melanoma and many cancer cell types [41]. The level of glypican-3 and survivin in cell lysate was elevated after test tumor cells were treated with colchicine, CMQ, FMQ or doxorubicin (Figure 1D). These results suggest that the three MDAs increase the expression of DAMPs and the expression of tumor-associated antigens such as survivin and glypican-3, but the patterns of these effects may differ among the MDAs and doxorubicin.

### Microtubule-depolymerizing agents can enhance the efficacy of therapeutic immunity provided by tumor cell lysate-pulsed dendritic cell vaccines

**Figure 2:**
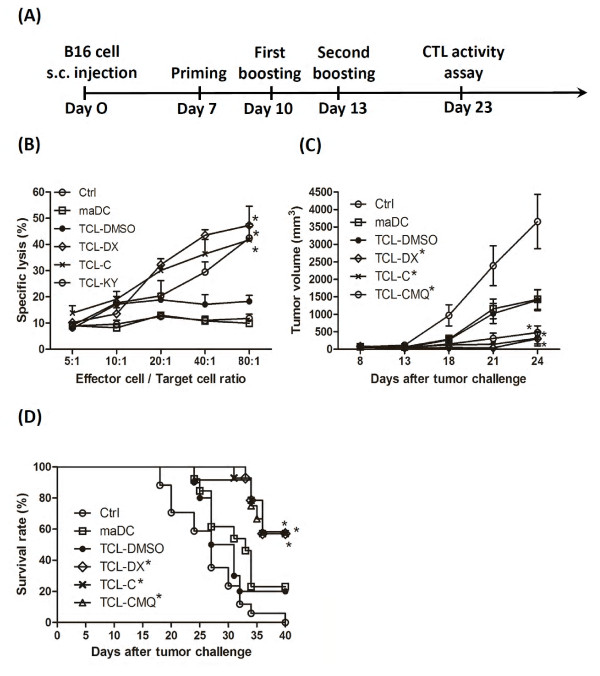
**Therapeutic immunity of DC vaccines pulsed with various tumor cell lysates, against B16 melanoma**. **(A) **Schematic representation for vaccination. C57BL/6 mice were challenged subcutaneously with 1 × 10^5 ^B16 cells and vaccinated with different preparations of TCL-pulsed DCs when the tumor volume reached to 50-80 mm^3^. On day 10 after the secondary boosting, splenocytes were harvested for cytotoxic T lymphocyte activity (CTL) assay. **(B) **CTL activities in test mice. Splenocytes from vaccinated or control group mice (*n *= 8 mice per group) were collected and CTL activity assayed with target tumor cells. Test DCs were pulsed with TCL-DMSO, TLC-DX, TLC-C, or TCL-CMQ. PBS-treated mice (Ctrl) did not receive DC vaccine, only a PBS injection. Test mice in the mature DC group (maDC) were subjected to vaccination with unpulsed mature DCs. **(C) **Tumor growth of treated mice. Tumor size of each group (*n *= 8 mice per group) was measured on indicated days. **(D) **Survival rates of treated mice. Survival of mice (*n *= 12 mice per group) was observed after tumor challenge. For all experiments, the TCL-FMQ groups showed a similar pattern of activities as that of TCL-CMQ and hence the data are not shown. All data are expressed as mean ± S.E.M.

The results described above suggest that MDA-conditioned TCLs may induce an immunogenic response *in vivo *and thus augment the efficacy of specific dendritic cell-based vaccines. A mouse model challenged subcutaneously with B16 cells and immunized with test DC vaccines intratumorally was used to explore this possibility (Figure 2A). Mice receiving PBS only, unpulsed mature DCs, or TCL-DMSO-pulsed DCs showed no significant cytotoxic T lymphocyte (CTL) activity against B16 melanoma (Figure 2B). However, mice vaccinated with TCL-C-pulsed DCs (*P *= 0.013 versus TCL-DMSO group) and TCL-CMQ (tumor cell lysate treated with CMQ) -pulsed DCs (*P *= 0.015 versus TCL-DMSO group were found to have significantly enhanced CTL activity with the ratio of effector cells to target cells (E:T) increased from 20:1 to 80:1. These CTL activities were comparable with those of the positive control, TCL-DX-pulsed DC vaccine (*P *= 0.018 versus TCL-DMSO group).

With respect to tumor growth, time-course experiments revealed that tumor growth was significantly inhibited in the TCL-DMSO (*P *= 0.02 versus vehicle group) in comparison with the unpulsed DC (*P *= 0.02 versus vehicle group) and vehicle group on day 24 post tumor inoculation (Figure 2C). However, treatment with TCL-C (*P *= 0.011 versus TCL-DMSO group) and TCL-K (*P *= 0.020 versus TCL-DMSO group) showed significant tumor suppression, which was comparable to that of the positive control, TCL-DX (*P *= 0.012 versus TCL-DMSO group). Furthermore, the survival time and survival rate of test mice in the TCL-C, TCL-CMQ and TCL-DX vaccinated groups (all with *P *< 0.001) were significantly improved in comparison with those of the TCL-DMSO group (Figure 2D). This trend was in accordance with that for the suppression of tumor growth (Figure 2C). Results for mice vaccinated with TCL-FMQ-pulsed DCs were similar to those vaccinated with TCL-CMQ pulsed DCs (data not shown). These results suggest that the efficacy of DC-based vaccines pulsed with TCLs can be effectively elevated by treating test tumor cells with the MDAs, colchicine, CMQ and FMQ.

### Specific immune cell subsets involved in vaccine efficacy

**Figure 3:**
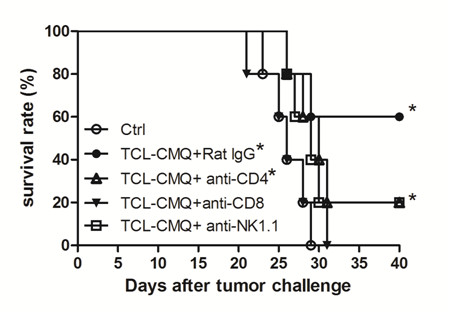
**Determination of immune cell subsets responsible for protective immunity induced by test DC vaccines**. Preparations of anti-CD4 (GK1.5), anti-CD8 (53-6.7), and anti-NK1.1 (PK136) antibodies were administered by intraperitoneal injection on day 1 in test mice prior to vaccination on days 2, 5 and 8 after tumor challenge as described in Materials and Methods. C57BL/6 mice (n = 5) were immunized intratumorally with test DC vaccines on days 7, 10 and 13 after tumor challenge as described in Materials and Methods and inoculated with B16 melanoma cells (10^5 ^cells/50 μl/mouse) on day 0. Statistical difference was calculated by log-rank (Mantel-Cox) test for mouse survival and P values of less than 0.05 were considered significant as compared with control group (*, *P *< 0.05).

In order to investigate which immune cell subsets play roles in therapeutic immunity of test DC vaccines, mice were injected intraperitoneally with either anti-CD4, -CD8, or -NK1.1 monoclonal antibodies (mAb) to deplete the respective cell types. The vaccinated mice treated with rat-IgG (*P *= 0.028, versus control group) or anti-CD4 mAb (*P *= 0.049, versus control group) showed significantly increased survival rates and survival times as compared with the control group (Figure 3). However, the survival rate of the vaccinated mice injected with anti-CD4 mAb was 40% lower than the rat-IgG group. On the other hand, vaccinated mice treated with anti-CD8 or anti-NK1.1 mAb did not exhibit a statistical change in survival rate and survival time as compared with control group (*P *> 0.05, versus control group). Depletion of CD8^+ ^T cells and NK cells thus virtually completely blocked the protective activity of DC vaccines pulsed with CMQ-treated TCLs. These results indicate that tumor-specific CD8^+ ^T cells and NK cells play a crucial role in the observed therapeutic immunity induced by DC vaccines pulsed with CMQ-treated TCLs against B16 melanoma, whereas CD4^+ ^T cells are only partially involved in the antitumor activity.

### Effect of DC culture conditions on vaccines

**Figure 4:**
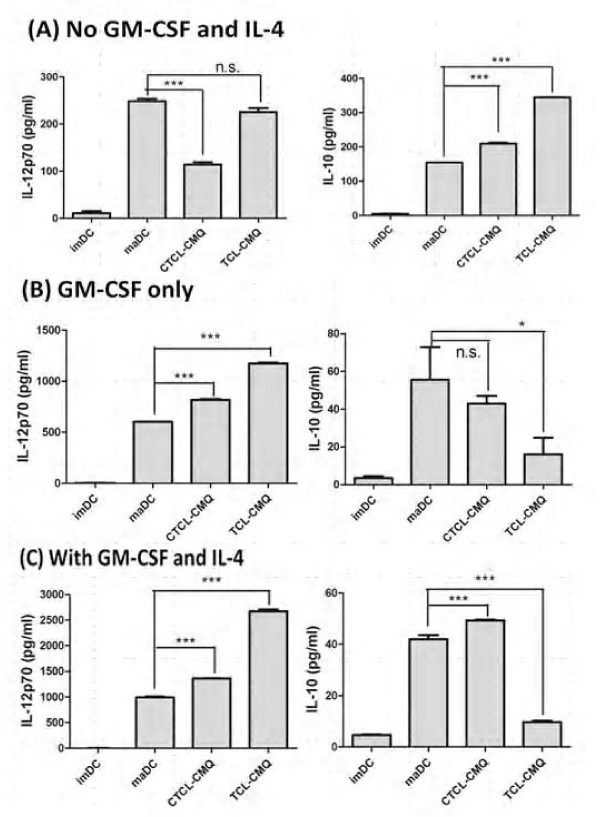
**Effect of GM-CSF and IL-4 on IL-12p70 expression in DCs treated with tumor cell lysates**. **(A-C) **IL-12p70 and IL-10 expression in DCs pulsed with CMQ-treated TCLs under different culture conditions. After harvesting, DCs were incubated in three kinds of culture conditions with or without GM-CSF (20 ng/ml) plus IL-4 (20 ng/ml), and were treated as follows: vehicle (imDC), LPS (maDC), TCL plus LPS for 24 h (CTCL-CMQ) or TCL for 12 h and then LPS for 24 h (TCL-CMQ) as described in Materials and Methods. The level of IL-12p70 released into supernatants of test DC cultures was assayed by ELISA. Statistical difference among test groups was analyzed by Student's *t*-test. P values less than 0.05 were considered statistically significant (*, *P *< 0.05; **, *P *< 0.01; ***, *P *< 0.001 versus the maDC group).

Since IL-12p70 plays a critical role in the effective priming of a T_H_1 anti-tumor immune response and CTL activities [26,42], and IL-10 has been shown to inhibit the production of IL-12p70 in murine DCs [43,44], we next evaluated the expression and balance of IL-12p70 and IL-10 activities in various TCL-pulsed DC vaccines. To address the possible effect of CMQ on test DCs, we determined the release of IL-12p70 and IL-10 expressed by murine DCs pulsed with or without CMQ-treated TCLs. Experiments were carried out simultaneously by co-treatment or co-incubation for 12 hours and stimulation with or without LPS in the absence of GM-CSF and IL-4 (Figure 4A). A higher production of IL-12p70 and a lower production of IL-10 were detected in DCs with LPS stimulation only (maDCs) than pulsed DCs co-cultured with TCL-CMQ and LPS (CTCL-CMQ) or with TCL-CMQ first and stimulated with LPS later (TCL-CMQ) (Figure 4A). DCs treated with TCL-CMQ showed increased production of IL-12p70 compared with imDCs and the CTCL-CMQ group, similar to the maDCs group, whereas the production of IL-10 was significantly increased in this group in comparison with other groups. The maDCs were more effective than TCL-CMQ and CTCL-CMQ in increasing production of IL-12, whereas TCL-CMQ was more effective than CTCL-CMQ and maDCs in increasing production of IL-10. Overall, these results suggest that it may be possible to improve IL-12 production and decrease IL-10 production to enhance anti-tumor activity of TCL-CMQ.

Treatment with IL-4 accompanied by GM-CSF can promote IL-12p70 expression and inhibit IL-10 expression in murine or human DCs [45-47]. We, therefore, hypothesized that adding GM-CSF and IL-4 to test culture during the pulsing and stimulation period may improve the balance in production of IL-12p70 and IL-10. We therefore cultured DCs without either GM-CSF or IL-4, with GM-CSF only, or with GM-CSF and IL-4 to measure the production of IL-12p70 and IL-10 in test cells treated as mentioned above and shown in Figure 4A-C. Supplement of GM-CSF and IL-4 conferred the highest level of expression of IL-12p70 and lowest level of expression of IL-10 (Figure 4C) as compared with the other culture conditions (Figure 4A and 4B). The increase in IL-12p70 and decrease in IL-10 expression levels suggested that TCL-CMQ and CTCL-CMQ treatments could be greatly improved by supplement with GM-CSF only or by addition of both GM-CSF and IL-4 concomitantly. TCL-CMQ conferred the highest levels of IL-12p70 production and lowest levels of IL-10 in comparison with other test groups. These results suggest that TCL-CMQ treatment combined with culturing in GM-CSF and IL-4 may augment the efficacy of DC-based vaccines pulsed with TCLs.

### Effect of different administration routes on therapeutic immunity of DC-based vaccines pulsed with tumor cell lysates

**Figure 5:**
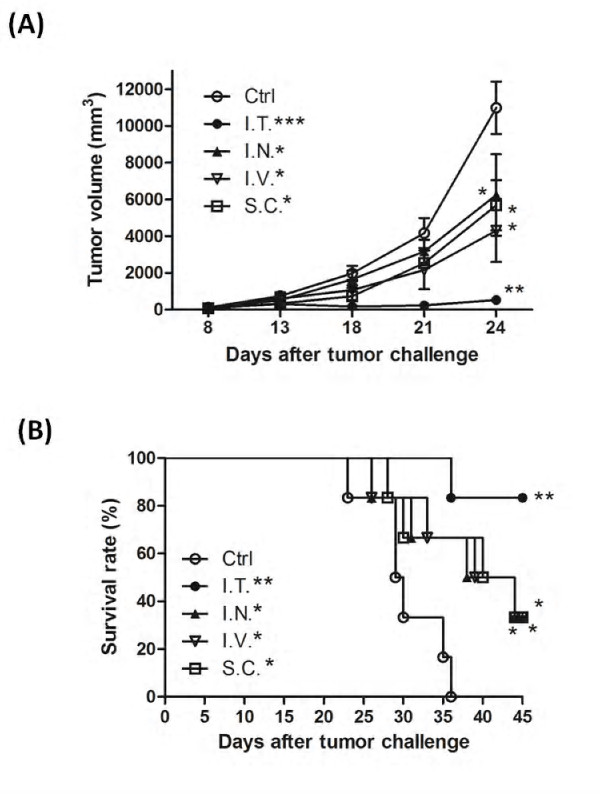
**Effect of different administrative routes on DC-based vaccines pulsed with specific tumor cell lysates**. C57BL/6 mice (*n *= 6) were immunized by intratumoral (I.T.), intranodal, (I.N.) or subcutaneous (S.C.) injection with DC-based vaccines pulsed with CMQ-treated TCLs on days 7, 10 and 13 after tumor challenge. One week post vaccination, right flanks of mice were subcutaneously inoculated with B16 melanoma cells (10^5 ^cells/50 μl/mouse). During the following 45 days post tumor challenge, tumor volume (**A**) and survival time (**B**) of mice was observed and measured as described in Materials and Methods. Analysis of statistical differences among test groups is described in Materials and Methods. P values of less than 0.05 were considered statistically significant (*, *P *< 0.05; **, *P *< 0.01; ***, *P *< 0.001).

IL-12p70 has been shown to be beneficial when used as an adjuvant with DC-based vaccines, either via systemic or local delivery, especially when administered via intratumoral injection [43,44,48]. It is clinically important to establish optimal methods of administration for effective delivery of TCL-pulsed DC-based vaccines. To evaluate of efficiency of vaccine delivery, test vaccines were injected intratumorally, intranodally, intravenously and subcutaneously into the left flanks of mice. Mice receiving intratumoral injection of TCL-CMQ DC vaccines (*P *< 0.01, versus control group) showed a stronger therapeutic immunity than all the other delivering systems tested (all *P *< 0.05, versus control group) with respect to tumor suppression (Figure 5A). Animal survival rate and time, as analyzed by log-rank (Mantel-Cox) test (Figure 5B), were also drastically increased by vaccination via intratumoral injection (*P *= 0.0011, versus control group) as compared to vaccination via intranodal injection (*P *= 0.0319 versus control group), intravenous injection (*P *= 0.0318, versus control group) or subcutaneous injection (*P *= 0.0355 versus control group). Nonetheless, it is important to note that the intranodal, intravenous and subcutaneous modes of vaccine delivery also had a significant effect on survival rate and time of test mice in comparison with non-vaccinated (control) mice. These results suggest that the DC-based vaccine pulsed with TCLs delivered by intratumoral injection may be an efficient experimental mode for laboratory animal and perhaps human clinical studies.

### Microtubule-depolymerizing agents enhance maturation of dendritic cells and CD4^+ ^T and CD8^+ ^T cell proliferation

**Figure 6:**
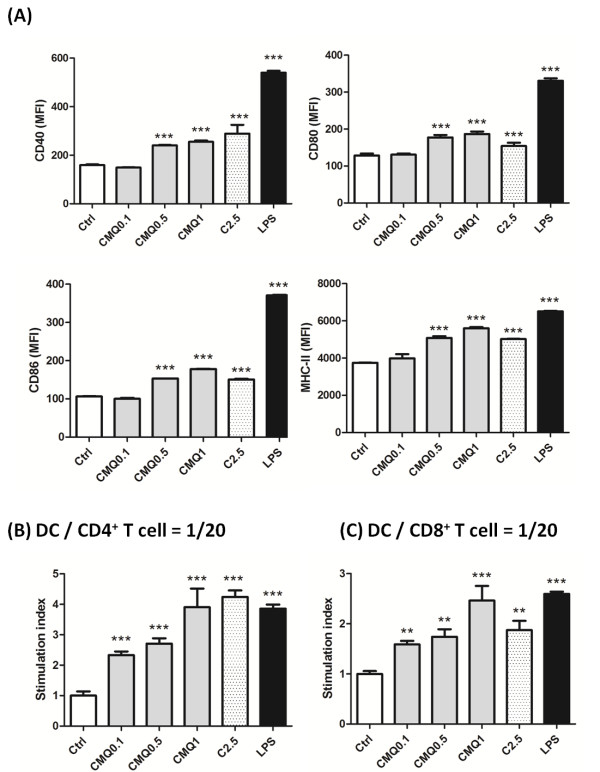
**Effect of treatment with different microtubule-depolymerizing agents on expression of cell-surface markers and CD4^+ ^and CD8^+ ^T-cell proliferation in mouse bone marrow-derived immature dendritic cells**. **(A) **Expression of surface markers on DCs. Cells were treated for 24 h with CMQ (0.1, 0.5 and 1 μM), colchicine (2.5 μM) and LPS (1 μg/ml), and harvested. Examination for the expression of CD40, CD80, CD86, MHC class II and CD11c markers was performed by flow-cytometry. The levels of CD40, CD80, CD86, MHC class II were expressed as mean fluorescence intensity (MFI). **(B) **CD4^+ ^and CD8^+ ^T-cell proliferation. After indicated treatments, treated DCs were co-cultured with CD4^+ ^T cells or CD8^+ ^T cells in a ratio of 1:20 (DCs versus CD4^+ ^T cells or CD8^+ ^T cells) for 72 h. T-cell proliferation was determined in vitro using a BrdU proliferation ELISA kit (Roche, Heidelberg, Germany) according to the manufacturer's instructions. The T-cell proliferation was expressed as the stimulation index, the OD_450 _value of co-culture of treated-DCs and T cells was divided by the value of co-culture of DMSO-treated-DCs co-cultured with T cells. All Data were expressed as mean ± S.D. P values less than 0.05 were considered statistically significant (*, *P *< 0.05; **, *P *< 0.01; ***, *P *< 0.001).

Previous studies reported that some microtubule-targeting agents including colchicine [15,16], vincristine [17] and paclitaxel [49,50] induced maturation of DCs and further augmented CD4^+ ^T and CD8^+ ^T cell activities or antigen cross-presentation activity. However, although 2-phenyl-4-quinolone derivatives have previously been shown to confer strong anti-tumor activity [32,36,51], whether these CMQ and FMQ phytocompound-derived chemicals could induce maturation of DCs and subsequently enhance CD4^+ ^T and CD8^+ ^T cell activities has not been reported. As a follow up, we treated DCs with various concentrations of CMQ (0.1, 0.5 and 1 μM), colchicine (2.5 μM) and LPS (1 μg/ml). As shown in Figure 6A, expression of DC maturation surface markers such as CD40, CD80, CD86 and MHC-II were greatly increased after treatment with CMQ as compared with those of control group cells. Levels of induction of DC maturation surface markers after treatment with CMQ were comparable to those observed for colchicine, but were less than those observed after LPS treatment. Co-cultivation of CD4^+ ^T or CD8^+ ^T with DCs showed that CMQ could significantly augment CD4^+ ^T and CD8^+ ^T cell proliferation (Figure 6B). Treatment with FMQ showed similar effects (data not shown). Our findings thus suggest that CMQ and FMQ can induce maturation of DCs and in turn enhance CD4^+ ^T and CD8^+ ^T cell proliferation.

## Discussion

The first therapeutic cancer vaccine, Sipuleucel-T (Provenge^®^), which uses antigen-presenting cell (APC) technology involving dendritic cells (DCs) for cancer immunotherapy was approved by the FDA in 2010 [52]. This success highlights the potential of *ex vivo *treated DCs as therapeutic vectors for various kinds of cell-based cancer vaccines. Immunization of cancer patients using their own DCs that have been loaded with tumor associated antigens (TSAs) and/or immune-modifiers *ex vivo *is becoming an increasingly popular strategy in the development of cancer vaccines [6,53]. Microtubule-depolymerizing agents (MDAs) such as colchicine and vincristine, which are used clinically in cancer chemotherapy, have recently been shown to enhance specific immune functions of DCs [15-17]. Whether or not MDAs such as these can be employed for use in DC-based cancer vaccines has, to the best of our knowledge, not previously been reported. To address this possibility, our current study explored the effect three MDAs - the well-known drug, colchicine, and two 2-phenyl-4-qunilone derivatives (CMQ and FMQ) - on immunogenic tumor cell death when used as "adjuvants" of TCL-pulsed DC vaccines in a therapeutic mouse model. MDAs were able to effectively induce the expression of immunogenic cell death-related proteins in targeted tumor cells, and augment the efficacy of TCL-pulsed DC-based vaccines.

Previous studies have shown that colchicine can elicit CD4^+ ^and CD8^+ ^T cell responses, induce antibody response, and promote antigen cross-presentation by murine dendritic cells [15,16,54,55]. In this study, we show that colchicine also induces the expression of DAMPs and tumor-associated antigens (TAAs) in dying B16 melanoma cells (Figure 1C-D). In addition, DC-based vaccines pulsed with colchicine-treated TCLs were able to enhance therapeutic immunity (Figure 2B-D). We also tested two 2-phenyl-4-quinolone derivatives, in parallel with colchicine, and doxorubicin (as a positive control), for their effect on this cancer vaccine approach. CMQ and FMQ showed effects comparable to colchicine; however, the detailed mechanism(s) of action of these MDAs remain unclear. Optimization of dosage and improvement in formulation of this MDA-TCL combination will be important for future use in DC-vaccines.

It has been put forward that DCs pulsed with TAA preparations derived from freeze-thaw cycle treatment of autologous TCLs are a promising approach to cancer immunotherapy as a wide repertoire of different TAAs are present in the lysate [25,26,28,56]. Based on knowledge of antigen-processing and cell trafficking of DC activities *in vivo*, it is generally believed to be important to optimize the *in vitro*/*ex vitro *culture conditions to generate efficacious vaccines for cancer immunotherapy. In cytokine-regulated anti-tumor immunity, IL-12p70 is recognized as a key cytokine in the promotion T_H_1 immune response [44]. IL-10 inhibits the releases of IL-12p70, and is often considered as unfavorable for promoting anti-tumor immunity. We showed here (Figure 4A-C) that supplement of GM-CSF only or GM-CSF plus IL-4 into the culture medium of a DC-based vaccine can drastically alter the balance of IL-12p70 versus IL-10 levels in TCL-pulsed, LPS-activated DCs. Hatfield et al. reported that DCs pretreated with TCLs and then activated by LPS treatment expressed a substantially reduced level of IL-12p70 and a substantially increased level of IL-10 [26]. Interestingly, here we show that supplement of the cytokines GM-CSF and IL-4 to the culture medium throughout the entire TCL-pulse/LPS-activation incubation period can drastically reverse such IL-12p70/IL-10 expression ratio (Figure 4A-C). We therefore believe that this protocol could increase the potency of DC vaccines for use in anti-tumor vaccination [26].

Studies on DC-vaccines against cancers have used a number of different modes of administration for delivery. Unfortunately, little or no comparative analysis of those delivery systems is available. For future potential clinical application, we consider that it is important to investigate which delivery system(s) is practical and desirable for such vaccines. Intratumoral injection of test vaccines elicited the best therapeutic effects among all tested administrative routes (Figure 5A and 5B) in this study. In cancer patients, DCs often present in an immature or dysfunctional state, especially tumor-infiltrating DCs, thereby preventing stimulation of tumor-specific T cells [57]. Our findings suggest that, for large size tumors, intratumoral injection of DC vaccines, may be the most efficacious delivery mode. However, intratumoral injection has several disadvantages including lack of promotion of systemic circulation, and inconvenience of delivery into tumor sites in some clinical tumors. Therefore combinations of different administration modes still need to be considered to achieve efficacy.

Previously we developed particle bombardment/gene gun technology, by which 0.1 to 1 million 1-3 μM gold particles (biologically inert) can penetrate into epidermal or dermal tissues [58]. We also reported that such ballistic bombardment can systematically generate evenly distributed, microscopic tissue tracks, creating mild, defined tissue and microvascular wounding in target skin areas [59,60]. We propose that anti-cancer vaccines may be optimized by making use of the particle bombardment technology as a systematic, highly effective and multiple site-delivery mode for the pretreatment of tumor-bearing mice areas (especially for the high number, metastasized, microscopic melanoma nodules), before administration of DC-based vaccines. In future possible applications, melanoma patients may be administered TCL-DC anti-cancer vaccines using a combination of gene gun and intratumoral injection of TCL-DCs

Apoptotic cell death was previously recognized mainly as being tolerogenic, whereas necrosis was considered immunogenic. Recently, it was found that apoptotic cell death also can induce immunogenic cell death via expression of DAMPs [9,10,23,39,61]. A number of studies have also shown that DCs pulsed with apoptotic tumor cells can induce immunity against tumors [23,61]. Whether human DCs pulsed with MDA-treated tumor cells could elicit a strong immunity against human cancers needs to be investigated in future clinical studies.

Regardless of which pathways are targeted by DC-based vaccines to treat tumor cells, apoptotic or necrotic, it is generally agreed that treatment needs to result in immunogenic cell death, especially in expression of DMAPs [8,38-40]. Currently, physical methods includingγ-irradiation [62], heat stress [26] and UV [63] are used to enhance expression of DMAPs to generate strong tumor-specific immunity. And chemotherapeutic agents including doxorubicin, mitoxanthrone, oxaliplatin have been shown to confer these activities [7,64,65]. Here, we show that MDAs can also induce immunogenic cell death and confer immunity against test tumors. We speculate that a combination of physical and chemical methods may help upgrade efficacy of either the TCL- or apoptotic tumor cell-pulsed DC vaccines. Future research is required to address these possibilities.

Recent studies have suggested that microtubules play a role in antigen-presenting presentation of dendritic cells [13,54]. An increasing body of evidence has also shown that microtubule-targeted agents including vincristine, colchicine and palitaxel can promote DC-mediated immune responses or enhance efficacy of combined chemotherapy [15-17,49,66]. The binding site of 2-pheny-4-quinolone derivatives on microtubule proteins was reported to be likely the same as the binding site of colchicine [32]. Our current findings revealed that 2-phenyl-4-quniolone derivatives can enhance the maturation of DC and mediate promotion of CD4^+ ^and CD8^+ ^T cell proliferation (Figure 6A and 6B). The specific microtubule-depolymerizing agents we studied here may thus have potential for use as adjuvants for DC-mediated vaccines for infectious diseases or melanoma cancers.

## Conclusions

Three specific microtubule-depolymerizing agents - colchicine, 2-(3-chlorophenyl)-6,7-methylenedioxyquinolin-4-one (CMQ) and 2-(3-fluorophenyl)-6,7-methylenedioxyquinolin-4-one (FMQ) - were found to effectively induce expression of DAMPs proteins and augment the therapeutic efficacy of DC-based vaccines pulsed with TCLs. In test B16 melanoma mouse systems, CD8^+ ^T cells and NK cells were found to be involved in the observed therapeutic immunity. Further, supplementing culture media with GM-CSF and IL-4 culture prior to and during pulsing of MDA-treated DCs may enhance the efficacy of vaccines. CMQ and FMQ were also able to induce maturation of DCs and increase CD4^+ ^T and CD8^+ ^T cell proliferation. Our findings showed conclusively that colchicines, CMQ and FMQ offer bifunctional anti-tumor protection in a B16 melanoma model in rodents: First, these chemicals upregulate tumor DAMP, and cause immunogenic cell death of tumors; second these MDAs strongly augment tumor antigen presenting function of mature DC, which in turn orchestrate CD4- and CD8-mediated tumor destruction. The synergy of immunogenic cell death at level of tumor cells, coupled with their subsequent adjuvant effect for tumor antigen presentation at level of DC lends significant import for cancer vaccine development. Thus our findings strongly suggest that specific microtubule-depolymerizing agents, especially colchicine, may be suitable for clinical applications as adjuvants in TCL-pulsed DC vaccines. In the future, clinical studies on patients with advanced melanoma should be considered.

## Competing interests

The authors declare that they have no competing interests.

## Authors' contributions

CCW and HMC served as the key experimenters and authors of the draft manuscript. SSC provided useful ideas for the draft manuscript. LTH helped western blot analysis for expressions of immunogenic cell death-related proteins. WTC, PA and WCW helped with preparation of dendritic cells-based vaccines and animal models to evaluate the effects of MDAs on therapeutic immunity against B16 melanoma. LCC and JBW synthesized the chemicals tested. NSY and SCK finalized this manuscript and are the principal investigators and corresponding authors of the manuscript. All authors read and approved the final manuscript.
